# Symbiotic cellulolytic bacteria from the gut of the subterranean termite *Psammotermes hypostoma* Desneux and their role in cellulose digestion

**DOI:** 10.1186/s13568-019-0830-5

**Published:** 2019-07-17

**Authors:** Huda R. K. Ali, Nada F. Hemeda, Yasser F. Abdelaliem

**Affiliations:** 1 Plant Protection Research Institute, A. R. C, Giza, Egypt; 2 Department of Genetics, Faculty of Agriculture, Fayoum University, El Faiyûm, Egypt; 3 Agricultural Microbiology Department, Faculty of Agriculture, Fayoum University, El Faiyûm, Egypt

**Keywords:** Termite, *Psammotermes hypostoma*, Symbiosis, Cellulose degrading bacteria, 16S rRNA gene, Phylogenetic analysis

## Abstract

The subterranean termite *Psammotermes hypostoma* Desneux is considered as an important pest that could cause severe damage to buildings, furniture, silos of grain and crops or any material containing cellulose. This species of termites is widespread in Egypt and Africa. The lower termite’s ability to digest cellulose depends on the association of symbiotic organisms gut that digest cellulose (flagellates and bacteria). In this study, 33 different bacterial isolates were obtained from the gut of the termite *P. hypostoma* which were collected using cellulose traps. Strains were grown on carboxymethylcellulose (CMC) as a sole source of carbon. Cellulolytic strains were isolated in two different cellulose medium (mineral salt medium containing carboxymethylcellulose as the sole carbon source and agar cellulose medium). Five isolates showed significant cellulolytic activity identified by a Congo red assay which gives clear zone. Based on biochemical tests and sequencing of 16s rRNA genes these isolates were identified as *Paenibacillus lactis*, *Lysinibacillus macrolides*, *Stenotrophomonas maltophilia*, *Lysinibacillus fusiformis* and *Bacillus cereus*, that deposited in GenBank with accession numbers MG991563, MG991564, MG991565, MG991566 and MG991567, respectively.

## Introduction

Termites are social insects occurring in tropical, subtropical, and temperate regions of the world. Presently, about 2800 species are known. Termites are regarded as harmful insects because of their ability to destroy all materials containing cellulose. Several species of wood-feeding termites are known for causing serious economic damage. In the United States, the costs for damage repair and termite control may reach up to 5 billion dollars per year (NPMA 2006).

The dangers of termites are known to most of people. Throughout the tropical regions everyone knows that termites are voracious eaters of houses, living trees and crops or any material contains cellulose, they can cause severe damage. In the USA termites cause more economic damage than flood combined with fire (Eggleton 2011). Predominantly by feeding on structural timbers they can use dead wood making them a major pest for timber used for construction purposes, both inside buildings and outdoors (Nobre and Nunes 2007).

In the USA, the presence of the destructive subterranean termite *Coptotermes formosanus* species can cost a multimillion-dollar termite control industry, therefore the tolerance threshold for termites is approaching zero in countries with a high relative standard of living (Su and Scheffrahn 1998). Eight species of subterranean termites were detected in Egypt. The subterranean termite, *P. hypostoma* Desneux is the most serious pest, causing damage to any materials containing cellulose, the annul control and repairing of damage costs millions of pounds, essentially in upper Egypt and the new valley (Ahmed et al. 2014). Depending on the termiticide sales in 2010, the world wide annually controls and repair cost was evaluated and data displayed that the global economic impact of termite pests has increased to $40 billion. Subterranean termites accounted for ≈ 80% of the costs ($35 billion) (Rust and Su 2012).

Termites can be divided into two categories: higher termites (Termitidae) and lower (all families except Termitidae) based on absence or presence of flagellate in their hindgut, respectively. Lower termites have many types of bacteria in addition to protozoa, while higher termites usually have only the bacteria and a more elaborate anatomy while lacking the protozoa (Varma et al. 1994; Eggleton 2011). It is also known that the higher termites degrade cellulose by using their own enzyme which were secreted by their gut and salivary glands (Breznak and Brune 1994; Ohkuma 2003; Ramin et al. 2008). Most termite gut flagellates are associated with bacterial symbionts. The bacteria can be found connected with the plasma membrane of the flagellates (ectosymbionts) and/or located in the cytoplasm or the nucleus of their hosts (endosymbionts). The symbiosis between the flagellates and bacteria seems to be highly specific (Stingl et al. 2005; Ohkuma 2008). Microbial communities in hindgut are associated with wood digestion in termite and play an essential role in supplementing this nutrient-poor food source. Symbionts stimulate reactions involved in the breakdown of all three major ingredients of wood (cellulose, hemicellulose, and lignin phenolics) and supplement this diet by synthesizing other essential nutrients (Husseneder 2010; Peterson and Scharf 2016).

Several strains of cellulolytic bacteria were isolated from the termite. Ramin et al. (2008) isolated three intestinal bacteria from the hind gut of the subterranean termite *Coptotermes curvignathus*, identified them as (*Enterobacter aerogenes, E. cloacae* and *Clavibacter agropyri*) and demonstrated their roles in cellulose degradation. Eight bacterial and five fungal cellulose degrading isolates were isolated from the termite gut, the isolated organisms were identified as *Bacillus* sp., *Cellulomonas* sp., *Enterobacter* sp., and *Aspergillus* sp. The Congo red screening assay for cellulase production showed the widest zone of hydrolysis (38 mm) for *Aspergillus* sp. (Sharma et al. 2015).

Sreena et al. (2015) isolated five effective cellulose degrading bacteria from the hind gut of the termites genus *Heterotermes* and *Odontotermes*, three of them belonged to *Bacillus* and one each to *Staphylococcus* and *Enterobacter* sp.

There are 2800 termite species, but few have been examined to determine their gut flora. Few cellulolytic-bacteria were isolated and identified from certain termite species because of the difficulty in isolating and cultivating a large number of gut microorganisms.

To the best of our knowledge this is the first study on bacteria that have mutualistic relationships with this species of termite. These bacterial isolates can be used in the effective and novel control of subterranean termite in the future research to minimize pesticides usage. Since most previous studies are studying termite control using pesticides.

This study aims to isolate and identify cellulolytic bacteria from the gut of *P. hypostoma* and to confirm their role in cellulose degradation, because the ability to disrupt this function has potential use for termite control.

## Materials and methods

### Termite collection and identification

The Subterranean termite was collected using cellulose traps according to the method of El-Sebay (1991) which were located in Al-Haraga village, Fayoum Governorate, Egypt on February 2017. The termite samples were then transported immediately to the microbiology lab and washed with tap water, to get rid of any adherent dirt. The termite was identified based on its morphological characteristic at Department of Termite and Wood Borers, Plant Protection Research Institute Agricultural Research Center, Giza, Egypt.

### Isolation of cellulase producing bacteria

One hundred termite workers were surface sterilized with 70% ethanol then washed with sterile distilled water. After cutting their heads using a syringe needle, the entire guts were removed and macerated on sterilized sand. Cellulase producing bacteria were isolated according to the method of Gupta et al. (2012), the guts samples were inoculated in broth mineral salt medium (NaNO_3_ 2.5 g; KH_2_PO_4_ 2 g; MgSO_4_ 0.2 g; NaCl 0.2 g; CaCl_2_·6H_2_O 0.1 g in a liter) containing 0.5% carboxymethylcellulose (CMC) as a sole carbon source, the pH of the medium is adjusted to 7.0. The inoculated flasks were incubated for 2 weeks at 37 °C. Subsequently, subculture was repeated using same broth mineral salt medium (MSM) and using the same conditions.

To select the cellulolytic bacteria and estimate the ability of the bacteria to digest cellulose, 1 mL of the culture was spread on cellulose agar plates medium composed of K_2_HPO_4_ (0.2 g/L), KH_2_PO_4_ (0.2 g/L), MgSO_4_ (0.2 g/L), NaCl (0.2 g/L), NaNO_3_ (1 g/L), CaCO_3_ (0.01 g/L), yeast extract (0.5 g/L), CMC (10 g/L), Agar (15 g/L), pH 7.0 and incubated for 48 h at 37 °C. All bacterial isolates were purified by re-streaking on CMC agar plates. The most promising cellulase producing isolates were selected for further investigation and characterization. Isolates were stored at − 80 °C in sterilized glycerol (20%).

### Screening for cellulolytic bacteria

The pure colonies from each isolates were grown in 50 mL medium containing (g/L) 0.2 K_2_HPO_4_, 0.01 MgSO_4_, 1 NaCl, 0.5 NaNO_3_, 0.05% yeast extract and 10 g CMC according to Sharma et al. (2015). Cultures were incubated at 37 °C for 72 h. At the end of incubation 1 mL of each culture suspension was centrifuged at 5000 rpm for 10 min and the pellets were re-suspended in fresh medium. Spotte and well diffusion assay method was used to estimate the cellulose degrading abilities of the isolates, 6 mm wells were punched in the 1.5% agar plates supplemented with 1% CMC. The wells were filled with 100 μL of each culture re-suspension prepared above and the plates were incubated at 37 °C for 24 h. The plates were submerged with 0.1% Congo red for 15 min and destined with 1 M sodium chloride solution for 15 min. The clear zones were measured.

### Enzyme activity assay

Cellulase (endoglucanase) activity in the supernatant was measured by the determination of reducing sugar released from carboxymethylcellulose (CMC) through dinitrosalicylic acid (DNS) according to Miller (1959). For comparison among all isolates, the same cell densities at 600 nm was used to eliminate the biomass effect on the evaluation. The amount of reducing sugar released in the supernatant was measured for treatments and control. One unit of cellulase was defined as the amount of enzyme which produces 1-μmol glucose equivalent per min under the assay conditions. The absorbance was measured with the visible spectrophotometer (Spectronics, India) at 540 nm. The amount of released reducing sugars was determined using a standard curve recorded for glucose.

### Identification and characterization of the isolates

#### Phenotypic and physiological characterization of the selected isolates

Before phenotypic and genotypic characterization of selected isolates, the purity was confirmed by streaking a single colony of isolates on nutrient agar as well as by microscopic examination. Preliminary morphological and physiological characterizations of the promising isolates were performed according to Gerhardt et al. (1994). The results were compared to those described in Bergey’s Manual of systematic bacteriology (Holt 2005).

### Molecular characterization of selected isolates

#### Isolation of genomic and amplification of 16S-rDNA gene

Genomic DNA was isolated by ABT genomic DNA mini extraction kit spin column (Applied biotechnology, Egypt) according to the manufactures instruction. To confirm the species of bacterial isolates at the molecular level, the 16S rDNA genes of the five isolates were amplified by PCR using the universal primers (reverse primer: 5′-GAGAGTTTGATCCTGGCTGGCTCAG-3′ and forward primer: 5′-AAGGAGGTGATCCAGCCGCA-3′) according to Cheng et al. (2010). The PCR was performed in the thermal cycle 2720 (Applied Biosystem, USA) in a total volume of 25 μL containing 1 μL of each primer, 3 μL of template DNA (50 ng/μL), 12.5 μL of 1× PCR master mix (GeneDireX) and 7.5 μL of water nuclease-free).

Amplification of 16S rDNA gene was executed under the following conditions: initial denaturation at 94 °C for 5 min followed by 35 cycles of 1 min denaturation at 94 °C, 1 min primer annealing at 52 °C, 1 min extension at 72 °C and final extension at 72 °C for 10 min. About 8 μL of PCR product for each samples were analyzed by electrophoresis on 2% agarose gel, previously stained with ethidium bromide (0.2 μg/mL) and were run at 120 V for 1 h and the amplicons were visualized under UV transilluminator.

#### Sequence analysis of 16S rDNA gene

The PCR products were purified using Montage PCR Clean up Kit (Millipore) following manufacture instructions to remove unincorporated PCR primers. The PCR products of approximately 1500 bp were subjected to sequencing through lab technology services located in Korea and performed at Applied Biosystems model 3730XL automated DNA sequencing system. The GenBank Accession numbers for the 16S rDNA gene sequence of *Paenibacillus lactis* (AFC1), *Lysinibacillus macrolides* (AFC4), *Stenotrophomonas maltophilia* (AFC3), *Lysinibacillus fusiformis* (AFC2) and *Bacillus cereus* (AFC5) isolates were MG991563, MG991564, MG991565, MG991566 and MG991567 respectively.

### Computational analysis (BlAST) construction of phylogenetic tree

The data of the nucleotide sequence of the 16S rDNA gene obtained from the five isolates were subjected to alignment with *P*. *lactis*, *L*. *macrolides*, *S*. *maltophilia*, *L*. *fusiformis* and *B*. *cereus* sequences available in GenBank database (http://www.ncbi.nlm.nih.gov/Blast) according to the method described by Thompson et al. (1994). Phylogenetic dendrogram was executed by the clustal w 2.1 multiple sequence alignment programs. The unweighted pair group method with arithmetic mean (UPGMA) was used to generate phylogenetic trees according to Kumar et al. (2004).

## Results

In this study, cellulose-degrading bacteria were isolated, characterized and identified from the *P. hypostoma* gut. These isolates were tested for their ability to digest cellulose.

Collected termite belong to the species of the subterranean termite *Psammotermes hypostoma* Deseunex (Isoptera: Rhinotermitidae) as identified based on their morphological characteristics. This species of termites causes severe damage to any material containing cellulose and causes large losses especially in the Upper, Central Egypt and the new valley Governorate, Egypt.

**Fig. 1:**
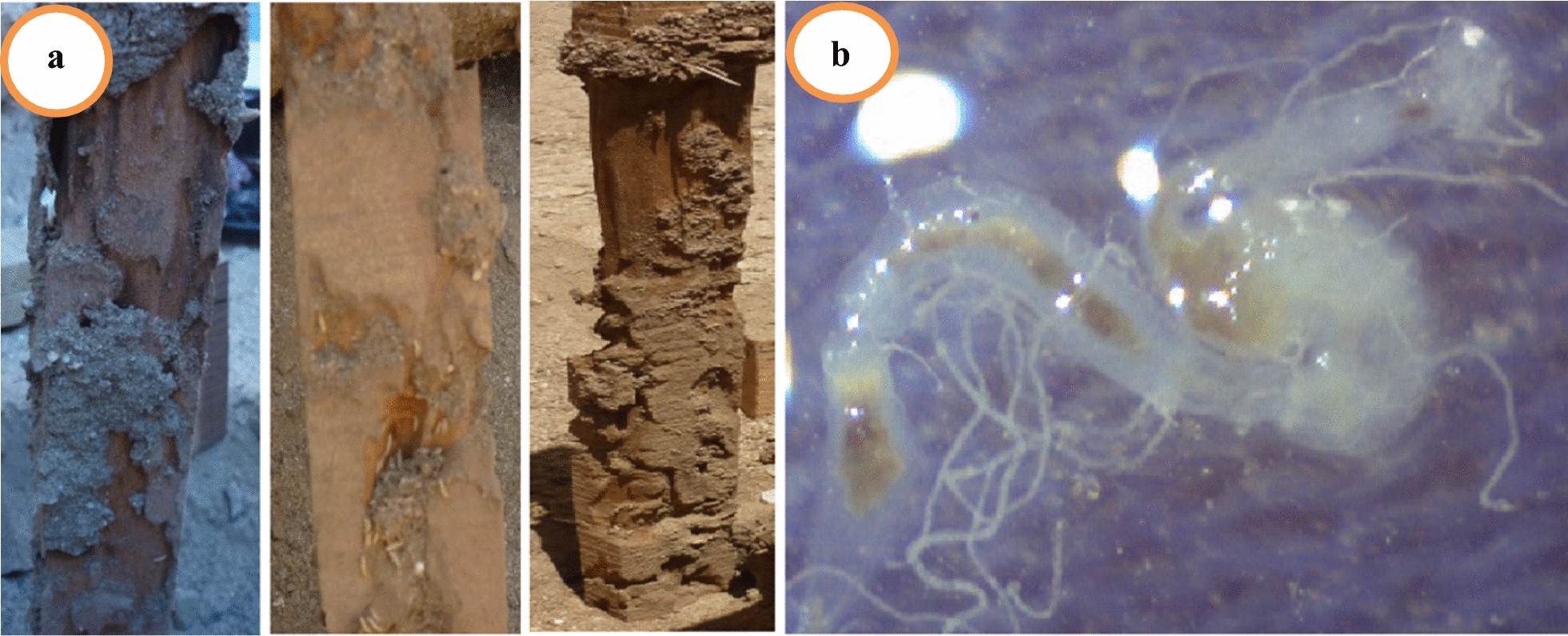
**a** Symptoms of wood damage by the subterranean termite, **b** gut of the subterranean termite *Psammotermes hypostoma* Desneux

**Fig. 2:**
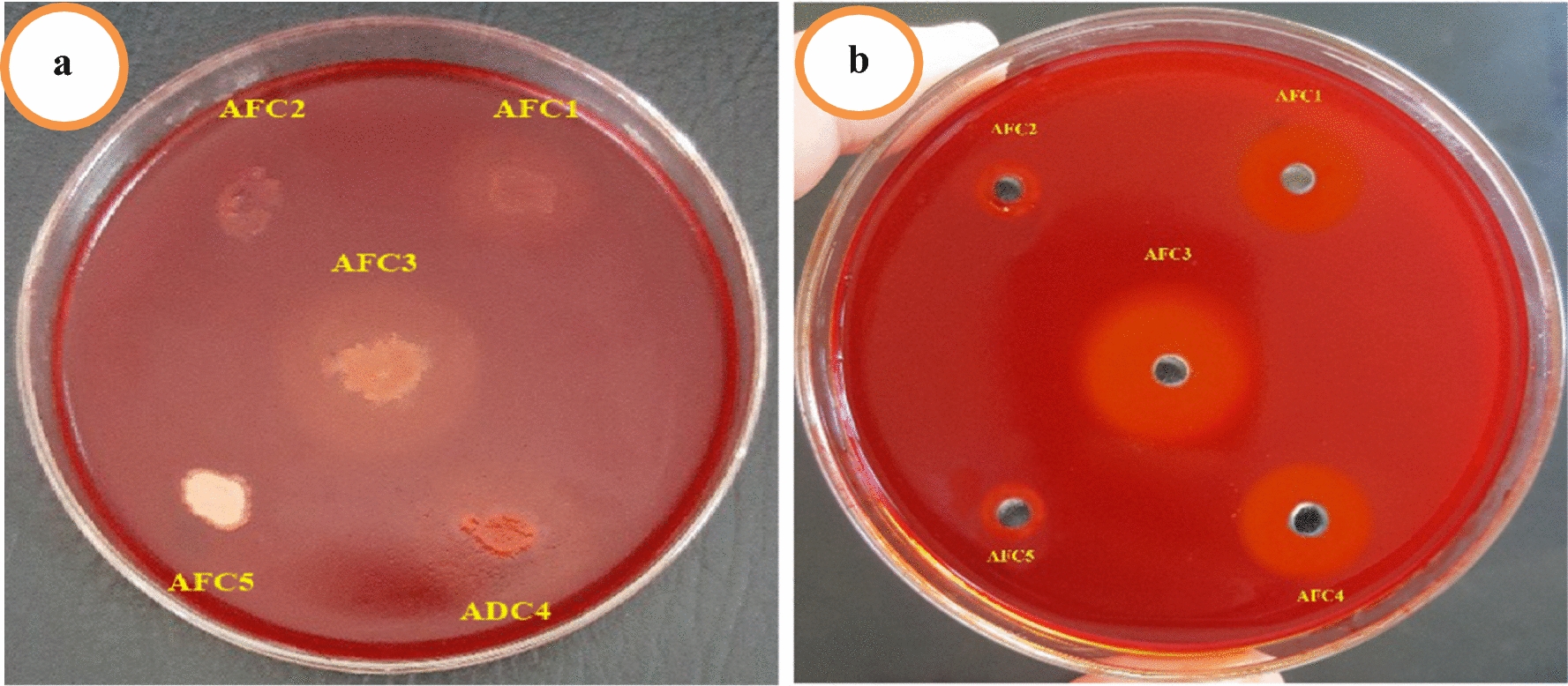
The ability of the isolates to degrade CMC by spotte (**a**) and well (**b**) diffusion assay method

The termite workers were dissected and their guts were obtained (Fig. 1), then the guts obtained from workers were macerated and inoculated in mineral salt media. Containing 0.5% CMC as sole of carbon source, 33 different isolates were obtained and all isolates were re-tested for purity and efficiency of enzyme production on CMC agar. Among the 33 isolates, only five bacterial strains were selected depending on its cellulolytic activity. Isolates showing considerable zone of clearance were characterized and size of the zone was recorded (Fig. 2).

Tables 1 and 2 shows the morphological and biochemical characteristics of the five selected isolates. Three isolates were identified as Gram-positive bacteria and two isolates were identified as Gram-negative bacteria. All isolates were able to grow in aerobic conditions. Isolate AFC3 was a motile and spore forming small rods, slightly tapered and curved with a circular greenish yellow colony on nutrient agar that identified as *S. maltophilia.* Isolates AFC2 and AFC4 were characterized as motile bacilli, while AFC5 was a motile and spore forming long rods, irregular with a brown colony on nutrient agar that identified as *B. cereus*. Isolate AFC1 was a motile and spore forming Long rods, slightly tapered and curved with a circular cream colony and greenish yellow colony on nutrient agar, indicating that AFC1 belonged to the genus *Paenibacillus*.

**Table 1:** Colony morphology of the bacteria isolated from termite gut contents

No.	Isolates	Color	Shape	Size (mm)	Elevation	Opacity
1	AFC1	Cream	Circular	1–2	Convex	Opaque
2	AFC2	White	Oval	1–2	Flat	Opaque
3	AFC3	Greenish yellow	Circular	3	Convex	Opaque
4	AFC4	Un pigmented	Compact	2–3	Convex	Opaque
5	AFC5	Brown	Irregular	3–4	Flat	Opaque

**Table 2:** Cell morphology of the isolated bacteria from termite gut contents

No.	Isolates	Gram stain	Size (µm)	Shape	Motile	Spore form	Oxygen requirements
1	AFC1	−	0.6 × 3.0	Long rods, slightly tapered and curved	+	+	Aerobic
2	AFC2	−	1.0 × 3.6	Short rods	+	+	Aerobic
3	AFC3	−	0.9 × 1.4	Slightly smaller and curved rods	+	+	Aerobic
4	AFC4	+	1.0 × 3.0	Short rods	+	+	Aerobic
5	AFC5	+	1.2 × 4.0	Long rods	+	+	Aerobic

Table 3 shows the cellulase (endoglucanase) activity for the five isolates was (1.47 ± 0.1 U/mL, 0.22 ± 0.1 U/mL, 2.28 ± 0.1 U/mL, 1.93 ± 0.1 U/mL and 0.23 ± 0.1 U/mL) for isolates (AFC1, AFC2, AFC3, AFC4 and AFC5), respectively. The results showed the highest isolate was AFC3 and the lowest cellulase activity for isolate AFC2. In this regard Gupta et al. (2012) reported the cellulase activity for eight bacterial isolates and found endoglucanase activity ranged from 0.162 to 0.400 IU/mL, however Sreena et al. (2015) isolated five cellulolytic bacterial strains from two termite species and found that the highest endoglucanase activity obtained for the isolate *B. cereus* strain ODO2 (5.06 U/mg), while the lowest endoglucanase for *B. cereus* strain ODO1 (2.98 U/mg). Also, Kamsani et al. (2016) measured the cellulolytic enzymes by microorganisms isolated from *Bulbitermes* sp. termite and found that *Bacillus* sp. B1 produced the highest endoglucanase (138.77 U/g) while *Brevibacillus* sp. Br3 produced the lowest endogucanase (3.46 U/g). Shinde et al. (2017) reported the highest cellulase (endoglucanase) activity by isolate C3 (*Klebsiella pneumonia*) was 0.41 (IU/mL) and the lowest activity by B2 isolate (*Klebsiella pneumonia*) was 0.21 IU/mL.

**Table 3:** Molecular identification of the five bacterial strains according to 16S rDNA gene sequence and their cellulase activity (U/mL)

Strain code	GenBank Accession no.	Bacterial name	CMCase (U/mL) at OD600 = 1
AFC1	MG991563	*Paenibacillus lactis*	1.47 ± 0.1
AFC2	MG991566	*Lysinibacillus fusiformis*	0.22 ± 0.1
AFC3	MG991565	*Stenotrophomonas maltophilia*	2.28 ± 0.1
AFC4	MG991564	*Lysinibacillus macrolides*	1.93 ± 0.1
AFC5	MG991567	*Bacillus cereus*	0.23 ± 0.1

### Phylogenetic analysis of the rDNA sequences

**Fig. 3:**
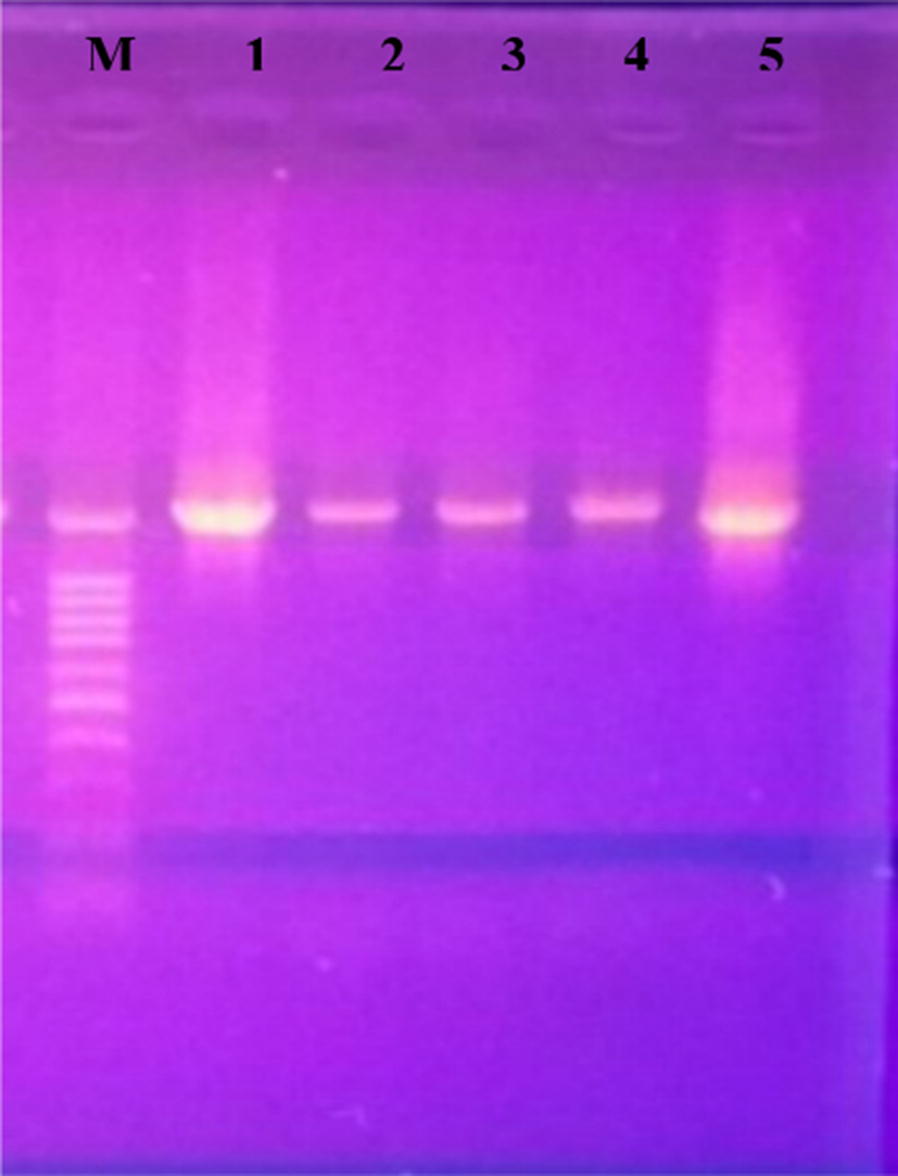
Agarose gel analysis of PCR products from amplification of 16S rDNA of five bacterial strains. Lane M: represents the molecular size marker (1 kb leader). Lane 1: represents *Paenibacillus lactis* AFC1 (MG991563), Lane 2: represents *Lysinibacillus fusiformis* AFC2 (MG991566), Lane 3: represents *Stenotrophomonas maltophilia* AFC3 (MG991565), Lane 4: represents *Lysinibacillus macrolides* AFC4 (MG991564) and Lane 5: represents *Bacillus cereus* AFC5 (MG991567) respectively

Amplification of the rDNA gene with universal primers revealed single product estimated by agarose gel electrophoresis approximately 1500 bp was obtained from all PCR amplification for five bacterial isolates (Fig. 3). Each sequence after editing was submitted to the GenBank and homology searching were done against the published bacterial sequences to compare with the related sequences in the GenBank using BLAST and FASTA programs. Search results of each sequence giving the closet match to the sample was used to determine the species of bacterial isolates.

The results showed that two isolates (AFC2 and AFC4) belong to the genus *Lysinibacillus*, and the other three isolates (AFC1, AFC3 and AFC5) belong to *Paenibacillus*, *Stenotrophomonas* and *Bacillus*, respectively (Table 3).

**Fig. 4:**
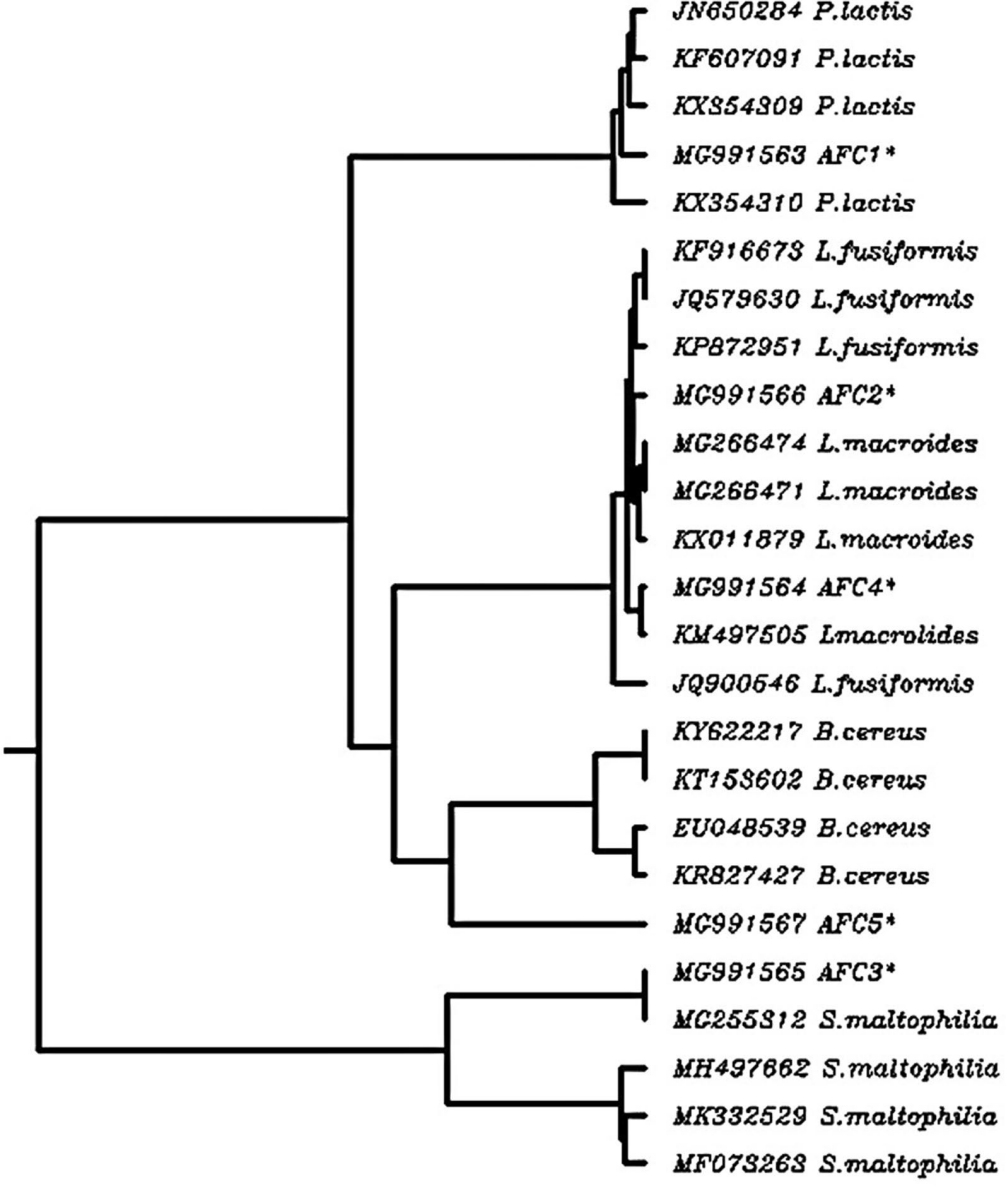
Phylogenetic tree showing the relationship between the five strains and the closely members of bacterial strains obtained from NCBI database according to 16s rDNA gene sequence

The phylogenetic tree obtained by sequence analysis of 16s rDNA of the five bacterial strains and the sequences of 20 closed bacterial strains obtained from GenBank database were represented in Fig. 4. According to the NCBI BLAST search of the sequence of the five isolates against the sequence of 20 other bacterial strains, the five isolates (AFC1, AFC4, AFC3, AFC2 and AFC5) were identified as *P. lactis*, *L. macrolides*, *S. maltophilia*, *L. fusiformis* and *B. cereus*, with Accession numbers (MG991563, MG991566, MG991565, MG991564 and MG991567), respectively (Fig. 4). |MG991563| AFC1 alignment scored 97.82% with *P. lactis* |JN650284|, |MG991564| AFC4 alignment scored 99.79% with *L. macrolides* |KM497505|, |MG991565| AFC3 alignment scored 100% with *S. maltophilia* |MG255312|, |MG991566| AFC2 alignment scored 99.30% with *L. fusiformis* |KP872951| and |MG991567| AFC5 alignment scored 98.65% with *B. cereus* |KY622217|.

## Discussion

This study allowed to unveil the role of some prokaryotes located in the hind gut of the subterranean termite *P. hypostoma* and confirm their principle role in cellulose digestion and nutrition. Another important role of the aerobic cellulolytic bacteria in the termite gut may be the consumption of oxygen and the creation of appropriate anaerobic conditions for the symbiotic obligatory anaerobic microorganisms (protozoa) that are indispensable for the digestion of consumed cellulose by the termites (Brune et al. 1995; Brune 1998; Butera et al. 2016).

On the other hand, microorganisms are considered the most important sources for enzymes production. Selection of the right organism plays a key role in high yield of desirable enzymes. Based on the above, *P. hypostoma* can be considered an acceptable source of cellulase enzyme for application in the bioethanol production.

In this regard, few cellulose degrading bacteria such as *Streptomyces*, *Acinetobacter, Pseudomonas, Bacillus, Ochrobactrum*, *Clostridium*, *Cellulomonas*, *Paenibacillus*, *Brevibacillus*, *Eubacterium*, *Serratia*, *Citrobacter*, and *Klebsiella* have been isolated and identified from some termite species (Schäfer et al. 1996).

Bacillaceae family has many cellulolytic members (Ito 1997), AFC2, AFC4 and AFC5 isolates belong to it. Other studies also frequently reported the isolation of cellulolytic bacilli from termites (Mathew et al. 2012; Kamsani et al. 2016). Also Peterson (1995) isolated three bacterial strains belonged to the *Bacillus* genus from the termite *Zootermopsis angusticollis*, and demonstrated their role in cellulose digestion.

All identified isolates were able to grow in aerobic conditions and selected by the Congo red test. Upon further quantitative determination of cellulose degrading enzyme, only five isolates displayed activity of cellulase (CMCase) between 0.22 and 2.28 U/mL with the highest enzyme activity demonstrated by the isolate AFC3. Table 3 shows all identified strains, their accession numbers and cellulase activity at OD600 = 1. Some biochemical parameters of these strains were investigated and the results were compared with Bergey’s Manual of Systematic Bacteriology (Holt 2005) (Table 4).

**Table 4:** Biochemical characterizations of isolated bacteria from termite gut

No.	Characterizations	Isolates				
		AFC1	AFC2	AFC3	AFC4	AFC5
1	Zone diameter (mm)	22	10	31	25	11
2	Oxidase	+	−	+	+	−
3	Catalase	+	+	+	+	+
4	Indole production	−	−	+	−	−
5	Methyl-red	+	+	+	−	+
6	Voges–Proskauer	−	−	−	+	+
7	Citrate utilization	−	+	+	+	+
8	Nitrate	±	−	−	+	+
9	Urease	−	+	+	±	±
10	Esculine	+	−	+	−	+
11	l-Arginine	−	−	+	+	+
12	Gelatin	−	+	+	±	+
13	H_2_S production	−	−	−	−	−
Sugar utilization						
14	d-Glucose	+	−	+	+	+
15	d-Lactose	+	−	+	−	−
16	d-Sucrose	−	−	+	−	−
17	d-Ribose	−	−	+	−	+
18	l-Arabinose	±	−	+	+	−
19	d-Mannitol	+	−	–	+	−
20	d-Sorbitol	−	−	–	−	−
21	Glycogene	+	−	–	−	+

Isolates AFC1 and AFC3 were identified as members of *P. lactis* and *S. maltophilia* respectively, while AFC4 was identified as *L. macrolides* and these findings are consistent with the findings reported by other workers in the same field (Schäfer et al. 1996; Peterson and Scharf 2016; Wenzel et al. 2002). Cellulolytic bacteria identified in our study belong to different bacterial genera.

The environmental microbial communities’ analysis has largely relied on a PCR-dependent amplification of genes entailing species identity as 16S rDNA (Rosselli et al. 2016). Butera et al. (2016) isolated cellulolytic bacteria from the gut of *Reticulitermes lucifugus,* demonstrated its role in cellulose digestion, and used 16S rRNA gene seq. to identify it. Also Wenzel et al. (2002) isolated 119 cellulolytic bacterial strains from the gut of the termite *Z. angusticollis* and used partial 16S rDNA seq. analysis to identify them.

In this study, we succeeded in isolating and identifying five strains of symbiotic an aerobic cellulolytic bacteria from the gut of the subterranean termite *P. hypostoma* indicating that they play a role in cellulose digestion in the termite gut as well as the cellulolytic flagellates and termite’s own cellulases enzymes. In addition to their role in creating anaerobic conditions, thus, the elimination of these aerobic cellulolytic bacteria deprives the termites from their role in cellulose digestion as well as altering the anaerobic conditions appropriate for the growth of symbiotic obligatory anaerobic protozoa (flagellate) to aerobic conditions leading to the death of the protozoa, thus eliminating a large part of the symbiotic organisms with termites, without which the termites can not live.

By disrupting mutual interactions between termite hosts and their symbionts, better management practices for these insect pests can be achieved in the next work.

## Data Availability

All data obtained have been included into the manuscript. Data scope and type: Nucleic acid sequence, gene, genome; Database: GenBank (National Center for Biotechnology Information). Strains deposition: The five strains were available upon request. The most efficient cellulose degraders were deposited in SCUF under the following numbers: AFC1 = SCUF01200; AFC3 = SCUF01201; AFC4 = SCUF01202
